# Dual Endothelin Receptor Inhibition with Bosentan Does Not Prevent the Early Formation of Post-Traumatic Joint Contracture in a Rat Model

**DOI:** 10.3390/jcm14196975

**Published:** 2025-10-01

**Authors:** Erik Wegner, Dennis Warnke, Victoria Buschmann, Benedikt Hild, Alexander Pirkl, Ulrike Ritz, Austin Harper, Erol Gercek, Philipp Drees, Andreas Baranowski

**Affiliations:** 1 Biomatics Group, Department of Orthopaedics and Traumatology, University Medical Center of the Johannes Gutenberg University, 55131 Mainz, Germany; 2 School of Medicine, St. George’s University, True Blue, St. George, Grenada; 3 Department of Trauma Surgery, Orthopaedics and Reconstructive Surgery, ANregiomed Hospital, 91522 Ansbach, Germany

**Keywords:** Bosentan, arthrofibrosis, joint contracture, trauma, translation, myofibroblast, PTJC

## Abstract

**Background:** Post-traumatic joint contracture (PTJC) remains one of the most prevalent and challenging complications arising from musculoskeletal trauma or surgical intervention. Conventional treatment modalities are largely reactive and address symptoms after onset, yet provide limited efficacy once contracture has developed. In contrast, pharmacological strategies targeting the underlying inflammatory and fibrotic pathways offer a promising strategy for preventing the development of PTJC altogether. **Methods:** A total of 26 male Sprague Dawley rats underwent standardized knee trauma followed by immobilization for a duration of two weeks. Rats were randomized into two groups. The experimental group (*n* = 13) received bosentan at a dosage of 50 mg/kg twice daily throughout the immobilization period. The control group (*n* = 13) received a placebo instead. Joint mobility was quantitatively assessed by measuring the contracture angle (CA) and resistance to extension. In addition, posterior joint capsule tissues were harvested for histological analysis and subjected to quantitative PCR (qPCR) to quantify the expression of profibrotic genes, including *α-Sma, Il-6, Tgf-β1, Nfκ-b, Ctgf*. **Results:** Bosentan had no relevant effect on the biomechanics of the contracture compared to the placebo group. The contracture angle was comparable between the groups (86.8° ± 14.1°, 84.8° ± 11.1°). Similarly, the force required to achieve knee joint extension was comparable between the groups. Gene expression analysis also provided no evidence of reduced expression of pro-inflammatory or profibrotic genes. Histological assessments revealed no change in the absolute or relative number of myofibroblasts, or in the number of vessels, in the posterior joint capsules of the rats treated with bosentan. Compared to the control group, the number of myofibroblasts significantly increased in both the bosentan and control groups (*p* < 0.001, one-way ANOVA). **Conclusions:** Bosentan’s purported antifibrotic properties do not appear to confer a preventative effect on the development of PTJC. These findings suggest that, despite its potential in modulating fibrosis, bosentan does not mitigate the progression of the fibrotic condition. Furthermore, the involvement of endothelin-1 (ET-1) in the pathophysiology of PTJC remains yet to be fully understood, warranting further investigation.

## 1. Introduction

The complete replacement of damaged tissue is fundamental to the restoration and preservation of physiological organ integrity. These self-limiting, homeostatic processes are mediated by a complex array of organ-specific cells, cells of the immune system, and their associated cytokines and mediators [1,2]. Even minor disruptions to this extremely precise interplay can lead to dysregulation, converting the normal physiological restoration process into a chronic pathological state. This can result in the progressive accumulation of extracellular matrix (ECM) and can affect the entire organ architecture via microstructural changes. This chronic pathological process, which can affect virtually any tissue, is commonly referred to as fibrosis. The irreversible loss of organ function marks the end stage of fibrosis and, depending on the system affected, can lead to a potentially life-threatening condition. If fibrosis affects the joints, it is referred to as arthrofibrosis [3]. Arthrofibrosis is characterized by an excessive deposition of extracellular matrix. It is accompanied by periarticular adhesions and may lead to chronic pain and significant restrictions in joint mechanics. The primary etiology of arthrofibrosis is severe or repetitive joint trauma, which may result from accidental injury but also includes invasive orthopedic joint surgery. In this context, the term post-traumatic joint contracture (PTJC) is also employed. Knee arthroplasty and cruciate ligament replacement are particularly prone to PTJC [4]. Consequently, PTJC is one of the most common complications following joint surgery and presents substantial challenges for both the patient and the surgeon [3,4]. Unlike other forms of organ fibrosis, even in its most advanced stages, PTJC does not significantly affect life expectancy; however, it does have a profound impact on the quality of life of those affected [3,5]. The degree of impairment is largely determined by the joint affected and the extent of the fibrosis. If a large joint such as the knee joint is affected, even the slightest restriction of range of motion (ROM) can lead to a disproportionately high degree of impairment [4,6]. In the absence of effective alternatives, the standard of care has remained unchanged for decades, consisting of lengthy physiotherapeutic mobilizations and mobilization under anesthesia. As an ultima ratio, the fibrotic adhesions can be surgically resected. However, the outcomes of these interventions are often inconsistent, as they neither prevent disease progression nor address the underlying pathology. Moreover, their invasive nature carries a significant risk of inducing fibrotic relapse [5,7]. Given these limitations, a pharmacological approach aimed at correcting dysregulated cell signaling cascades may be more effective.

**Figure 1:**
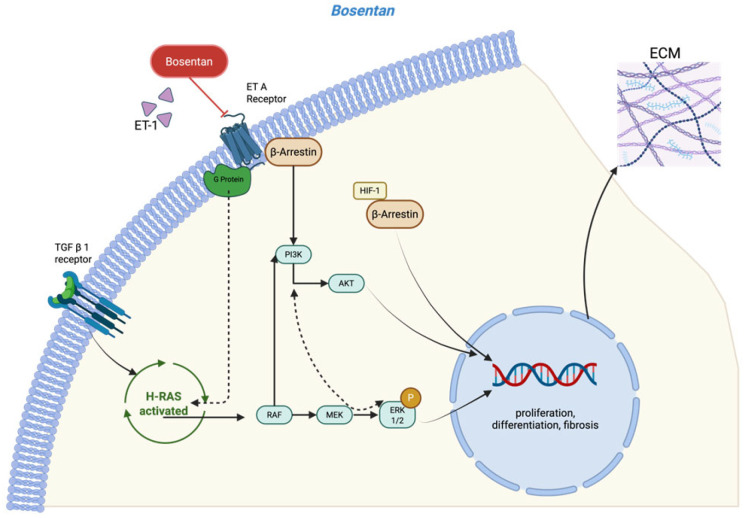
Model for the profibrotic signaling pathway involved in ET-1 and TGF-β and the role of the dual endothelin receptor antagonist bosentan [13,14] (created in https://BioRender.com).

Endothelin-1 (ET-1) plays a pivotal role in inflammation and fibrosis. It has been implicated in the pathogenesis of arthrofibrosis, with elevated tissue levels of ET-1 associated with a greater fibrotic response [8,9,10,11]. This peptide hormone, which is present in many cells of the immune system and fibroblasts but also synoviocytes, exhibits a high affinity for both endothelin receptor A (ETAR) and endothelin receptor B (ETBR) [8,12]. ETAR and ETBR are expressed on a variety of cell types. In fibroblasts, receptor activation induces collagen type I/III and fibronectin production, while inhibiting extracellular matrix (ECM) degradation enzymes and allows for fibroblast-to-myofibroblast transformation (FMT) [13,14]. As a G-protein-coupled receptor, ETAR/ETBR mediates ET-1’s profibrotic effect via the Rac/PI3K/Akt-dependent signaling pathway. In addition, ET-1 also acts as a co-factor in the TGF-β signaling cascade via the same receptor by activating Ras-Raf-MEK via the Nuclear Factor kappa-light-chain-enhancer of activated B cells (NF-kB) (Figure 1). Bosentan, a competitive antagonist of both ETAR and ETBR, has been approved for the treatment of various forms of pulmonary arterial hypertension. In animal models of cardiac, renal, and pulmonary fibrosis, bosentan has demonstrated a protective effect against ET-1-induced fibrosis [10,15,16]. These findings suggest that early intervention with bosentan may offer therapeutic potential in preventing the onset of trauma-induced arthrofibrosis.

In conclusion, post-traumatic joint contracture represents a significant challenge in clinical practice. While traditional approaches have primarily revolved around rehabilitation and surgical interventions, investigating pharmacological treatments opens exciting new possibilities for both prevention and management of this condition. To assess the potential benefits of bosentan for PTJC, we conducted a randomized, placebo-controlled trial in a rat model following knee injury. The primary objective was to assess whether orally administered bosentan improves knee joint mobility at the onset of PTJC. Additionally, this study aimed to determine whether the agent reduces the number of myofibroblasts and vascular proliferation, as well as reducing the concentration of pro-inflammatory and profibrotic cytokines. The results of this study will aid in advancing drug-based preventive strategies for post-traumatic joint contracture.

## 2. Materials and Methods

### 2.1. Study Design

**Figure 2:**
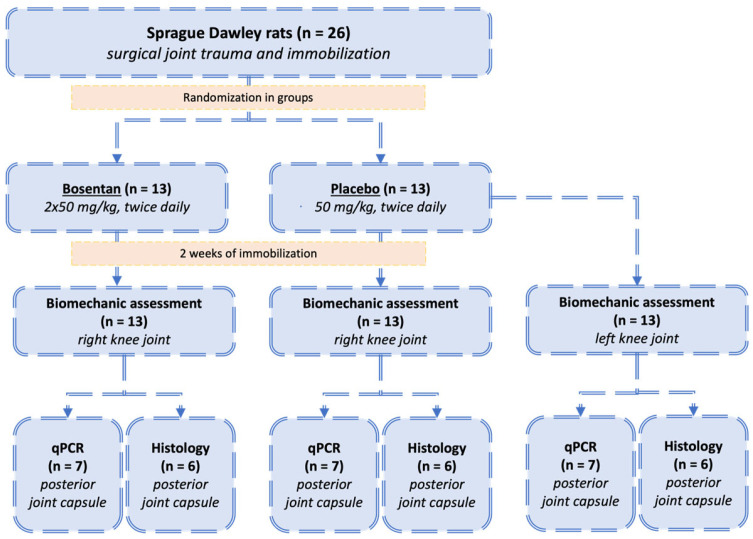
Study design: Allocation of animals to bosentan or placebo groups. All animals (*n* = 26) underwent the same surgical trauma and immobilization of the right knee joints. The animals were subsequently randomized into two groups: the bosentan group (*n* = 13) and the placebo group (*n* = 13). Following a 2-week immobilization period, biomechanical analysis was conducted. The posterior joint capsule was then designated for either histological evaluation (*n* = 6) or gene expression analysis (*n* = 7). Additionally, the uninjured left legs of the placebo group (*n* = 13) were analyzed, and their range of motion served as a reference for the physiological mobility of uninjured rat knees.

Male Sprague Dawley rats (*n* = 26) from Janvier Labs (Saint-Berthevin Cedex, France) served as the experimental subjects in this study. At 11 weeks of age, these animals presented a mean weight of 450 ± 25 g and were maintained individually, in controlled environments, with ambient temperature and standardized 12:12 h light–dark cycles. Nutrition consisted of commercial rodent feed and water provided ad libitum. The study design incorporated two experimental groups (*n* = 13 per group): a bosentan-treatment group and a placebo-control group. Statistical parameters for sample size calculation were established in collaboration with the Institute of Medical Biometrics, Epidemiology, and Computer Science (University of Mainz), employing a 5% significance level (α) with 80% power (β). Prior to surgical procedures, GraphPad’s “Random numbers calculator” (https://www.graphpad.com/quickcalcs/randmenu/ (accessed on 22 January 2023) facilitated randomized group allocation. Surgical intervention on day 0 comprised standardized posterior capsular trauma to the right knee joint in all animals, with subsequent Kirschner-wire (K-wire) immobilization. Control measurements were obtained from contralateral knees in the placebo cohort. The pharmacological protocol involved daily oral administration of either bosentan (50 mg/kg twice daily; Janssen Pharmaceutica N.V., Beerse, Belgium) or a placebo (50 mg/kg/ twice daily; Winthrop Arzneimittel GmbH, Frankfurt am Main, Germany) from the time of surgery until the study termination. Both were administered at 12 h dosing intervals (Figure 2). The drugs were ground in a mortar. They were then mixed with white chocolate cream. The amount of cream was 0.25 g per dose. This was then offered to the animals. The treat was preferred to normal food by the animals, and it was consumed immediately. The bosentan administration regimen is based on the drug’s antifibrotic effect observed in other rat models. However, no comparative data on the treatment of arthrofibrosis in this species are available [17,18,19]. Following a two-week period of immobilization, all animals were euthanized. The euthanasia procedure was executed via carbon dioxide asphyxiation. All experimental procedures were conducted in a double-blinded manner, and the study received approval from the relevant ethics committee (ID 23 177-07/G 21-1-113).

### 2.2. Rat Model and Operative Technique

Articular trauma was induced according to a previously validated protocol [20]. This experimental model reproduced complex joint injury, incorporating posterior capsular disruption, intra-articular hemorrhage, osseous damage, and temporary immobilization. All surgical procedures were conducted under strict aseptic conditions on the right knee of each animal. The injury mechanism involved hyperextension of the anesthetized rat’s right knee to 0°, inducing a controlled posterior capsular rupture. Concurrent with this maneuver, we created a precisely dimensioned osteochondral lesion (1.0 mm diameter, 3.0 mm depth) in the intercondylar region, while meticulously preserving articular cartilage and maintaining ligamentous structures. This carefully calibrated osseous injury effectively simulated intra-articular fracture conditions with a resultant hemarthrosis. Post-injury immobilization was achieved through K-wire fixation, with a contoured wire traversing the femoral–tibial junction to maintain the knee in 35° flexion. Lateral radiography (Faxitron MX-20 Cabinet X-Ray System) confirmed the intended flexion angle, proper wire placement, and excluded inadvertent fractures. After a standardized 2-week immobilization period, we removed all fixation hardware through the original surgical approach. Before doing so, another X-ray was taken to rule out any material failure or fractures and to confirm the flexion angle. The anesthetic protocol comprised initial induction with 1% isoflurane inhalation followed by maintenance using a subcutaneous combination of fentanyl (0.005 mg/kg), midazolam (4.0 mg/kg), and medetomidine (0.375 mg/kg). Anesthesia reversal employed flumazenil (0.2 mg/kg) and atipamezole (1 mg/kg). Perioperative analgesia consisted of tramadol (1 mg/mL) supplementation in drinking water initiated 3 days preoperatively and maintained for 7 days postoperatively. Prior to a range-of-motion assessment, we performed comprehensive periarticular myotomy to eliminate potential confounding effects from muscular tension. This standardized procedure involved systematic dissection and transection of all musculotendinous structures within a defined perimeter (10 mm proximal and distal to the joint line).

### 2.3. Joint Angle Measurements

**Figure 3:**
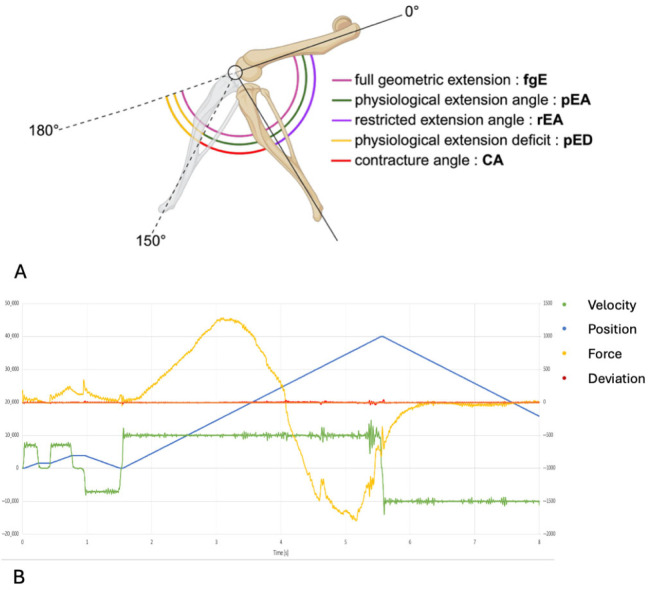
(**A**) The range of motion of a rat’s knee. The illustration depicts the joint angles of a healthy rat knee and a rat knee with post-traumatic joint contracture. A full geometric extension (fgE) is not possible because it is limited by the physiological extension deficit (pED). The remaining joint angles and their abbreviations can be found in the legend. (**B**) An exemplary representation of the measurement taken with the automated mechanical testing apparatus. “Force” is the product of the amperage used multiplied by a device-specific constant (1 N = 12 × 10 mA). “Position” indicates the joint angle. “Deviation” is the difference between the calculated internal position and the current motor position. “Velocity” is calculated using position and time.

**Figure 4:**
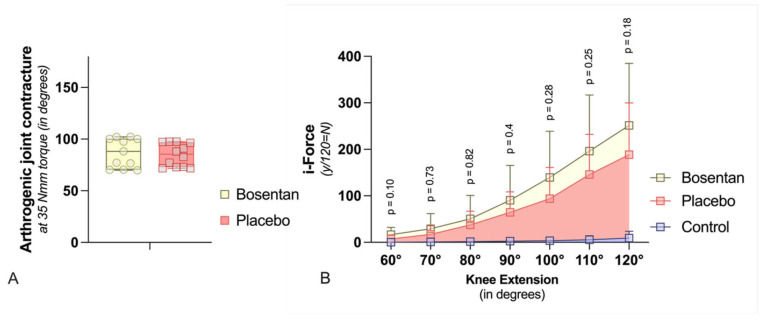
Biomechanic assessment (**A**) The graph compares the contracture angles of bosentan (*n* = 11) or placebo (*n* = 12) rat knees at a torque of 35 Nmm after 2 weeks of immobilization. The boxplot shows median (bar), maximum, and minimum (whiskers) values. There are no significant differences (Mann–Whitney U-Test). (**B**) In this graph, the effect of bosentan on biomechanical parameters is compared dynamically. The standard deviation is indicated by the whiskers. Bosentan (*n* = 11) does not reduce the force required to extend the joint from 60° to 120° compared to a placebo (*n* = 12). In addition, the force required for healthy knee joints (control, *n* = 13) is shown (multiple t-Test with Welch correction). Due to large variances, joint angles > 120° were not included.

The joint angle represents the angular relationship between femur and tibia. A fully extended knee joint (maximum range of motion) corresponds to an extension angle of 180° when these bones form a linear axis. Due to anatomical constraints, rat knees cannot achieve complete extension to 180°. Therefore, we defined a ”physiological extension deficit” as the difference between the maximal extension achievable in a healthy rat knee (at 35 Nmm torque) and complete joint extension (180°) (Figure 3). By definition, healthy rat knees exhibit only this physiological limitation rather than a pathological contracture. It has been demonstrated that a torque of 35 Nmm results in a full physiological extension of healthy knee joints without causing structural damage to the soft tissue of the joint [21,22,23]. We performed joint angle measurements in both experimental cohorts (bosentan and placebo). The contralateral (left) knees of the placebo group functioned as healthy controls since surgical interventions and immobilization procedures were confined to right knees (Figure 2). Contracture quantification involved calculating the difference between the mean extension angles of the control (left) knees and the measured extension angles of the corresponding right knees (Figure 3A). All measurements were conducted immediately post-euthanasia, two weeks after the initial surgical interventions. To isolate the arthrogenic component of contracture, the skin and periarticular musculature were completely transected at 1 cm from the femoral and tibial joint lines prior to measurement. For contracture evaluation, we developed a custom automated mechanical testing apparatus, modeled after previously validated systems for rabbit knee and rat elbow assessments [24,25,26] (Figure 3B). This apparatus incorporates a linear motor slide (Elax Ex 50F20, Jenny Science AG, Rain, Switzerland) with an actuator that applies controlled displacement while simultaneously measuring force via Forceteq force-displacement technology (Jenny Science AG, Rain, Switzerland). System control was maintained through a servo controller (XENAX Xvi Servo Controller with SMU (Safety Motion Unit) SS2 (Safe Stop 2), Jenny Science AG, Rain, Switzerland). The apparatus converts linear displacement into rotational motion through a rack-and-pinion mechanism, enabling load-controlled flexion–extension testing. Following limb secure fixation in specialized clamps, a single cycle extended the joint from its resting position toward a 180° extension angle. Force and torque measurements were continuously recorded throughout the testing cycles using Forceteq technology. Raw force-displacement data underwent conversion to torque and angular position parameters. Joint position was calculated algorithmically from linear motor stroke position. Contracture assessment was based on maximum extension measurements. The static and dynamic evaluation of the contracture were both calculated from the same cycle (Figure 4). The joint angle was determined when a torque of 35 Nm was reached in the test cycle for the static evaluation.

### 2.4. Tissue Preparation for Histological Analysis

Histological evaluation began with a careful dissection of the knee joints immediately following euthanasia. Sample distribution included the bosentan group (*n* = 6), the placebo group (*n* = 6), and unoperated control specimens from contralateral knees (*n* = 5). Tissue preservation involved initial fixation in a 4.5% neutral-buffered formalin solution (Carl-Roth, Karlsruhe, Germany) for 48 h. Subsequent decalcification was performed using a tris (hydroxymethyl)aminomethane (TRIS)-buffered 17.7% ethylenediaminetetraacetic acid (EDTA) solution (Applichem, Darmstadt, Germany). This process extended over six weeks at 20 °C with continuous agitation via a roller mixer to ensure uniform decalcification. Following standard laboratory protocols, the processed specimens underwent paraffin embedding. Central sagittal sections (5 µm thickness) were obtained from each joint. During quality control, sections were excluded that contained tissue-specific artifacts or structural damage to critical anatomical components, such as capsule tissue adjacent to the posterior meniscus base. The histological staining protocols included standard hematoxylin and eosin (H&E) for general morphometric assessment and vessel counting. Cellular phenotype identification was facilitated through *α-Sma* immunohistochemical staining according to previously established protocols detailed in our prior publications [27]. Digital image acquisition and morphometric analysis utilized ImageJ software (v. 1.54m). A total of nine high-power fields across three different slides per specimen were evaluated by two independent investigators under blinded conditions. Standardized quantification parameters included defined high-power fields (HPFs) measuring 62,500 µm^2^ (250 µm × 250 µm) positioned adjacent to the posterior meniscal margin. This consistent anatomical reference point ensured reproducibility across specimens. Myofibroblast quantification employed a differential analysis of *α-Sma* immunoreactivity with contextual interpretation. Specifically, *α-Sma* positive (+) cells were classified based on their spatial relationship to vascular structures. Those cells demonstrating perivascular localization were excluded from myofibroblast counts and categorized as pericytes or vascular smooth muscle cells [28]. The remaining *α-Sma* (+) cells distributed throughout the extracellular matrix were identified as differentiated myofibroblasts. Concurrent identification of *α-Sma* negative (-) cells within the extracellular matrix allowed a classification of the fibroblast and proto-myofibroblast populations. The vessels were enumerated in the same HPF as those utilized for the myofibroblast evaluation.

### 2.5. Tissue Preparation for Quantitative PCR

The posterior section of the knee joint capsule (*n* = 7 in the bosentan group, *n* = 7 in the placebo group, and *n* = 5 in the unoperated control/left knee) was immediately excised after euthanasia and immersed in RNAlater (Thermo Fisher Scientific, Waltham, MA, USA). It was stored at −20 °C until further processing for quantitative polymerase chain reaction (qPCR): Ribonucleic acid (RNA) was isolated by manually grinding the samples in liquid nitrogen, followed by additional homogenization using a Precellys homogenizer (Bertin Technologies, Montigny-le-Bretonneux, France) in a TRIzol suspension (Thermo Fisher Scientific), in line with the manufacturer’s instructions. The supernatants from the centrifuged homogenates were processed using a standard phenol–chloroform RNA extraction protocol (Sigma-Aldrich, St. Louis, MO, USA). RNA samples were suspended in nuclease-free water (Sigma-Aldrich) and quantified photometrically at 260 nm with a NanoDrop spectrophotometer (Thermo Fisher Scientific). Subsequently, 0.8 µg of RNA per sample was reverse-transcribed into complementary DNA (cDNA) using M-MuLV reverse transcriptase, a Random Primer Mix (New England Biolabs, Ipswich, MA, USA), and nucleotides (Bioron GmbH, Ludwigshafen, Germany). The primers for the qPCR (Table 1) were designed based on nucleotide sequences provided by the National Center for Biotechnology Information (https://www.ncbi.nlm.nih.gov/nucleotide/ (accessed on 20 June 2023), using a web-based primer design tool from the manufacturer (Eurofins Scientific, Luxembourg City, Luxembourg). The qPCR reactions were carried out on the qTOWER3 system (Jena Analytik, Jena, Germany) with the Blue S’Green qPCR Master Mix (Biozyme Scientific GmbH, Hessisch Oldendorf, Germany) as per the manufacturer’s instructions. ΔCt (Δ-cycle threshold) values were used for analysis, comparing the gene expression levels of *α*-*Sma*, *Il-6*, *Tgf-β1*, *Nfκ-b*, and *Ctgf* in the posterior joint capsule tissue.

### 2.6. Statistical Analysis

Statistical analyses were conducted using GraphPad Prism 10.3.1 (GraphPad Software, San Diego, CA, USA). Quantitative data are visualized through bar charts, box plots displaying medians and quartiles, mean values with standard deviations (mean ± SD), or area under the curve (AUC) representations. To assess joint contracture angles, the Mann–Whitney U test was employed, whereas histopathological data and gene expression data were analyzed using analysis of variance (ANOVA). Comparisons of gene expression levels were performed throughout, utilizing ΔCt values for statistical evaluation. All experimental measurements were performed in triplicate to ensure reproducibility. Statistical significance was determined at a threshold of *p* < 0.05. In the box plots, whiskers extend to the minimum and maximum values, the central line within the box denotes the median, and individual data points are represented as circles.

### 2.7. Graphic Illustrations

The schematic illustrations were created using the BioRender platform (https://bioRender.com).

## 3. Results

### 3.1. Perioperative Weight Development and Complications

At the beginning of this study, the average weight of the rats was 450 ± 25 g. No significant difference in weight development was observed in either the bosentan group or the control group. Both groups showed steady weight gain. After 2 weeks, the average weight in the bosentan group was 502 ± 31 g and in the placebo group it was 489 ± 24 g.

The X-ray examination prior to implant removal revealed a fracture of the femur proximal to the K-wire insertion in one animal in the placebo group. The surgically adjusted joint angle of 35° remained unchanged. An arthrometric measurement was not carried out on the fractured leg. Nevertheless, the posterior joint capsule was evaluated for qPCR and histology. One animal in the Bosentan group had to be removed prematurely due to wound dehiscence. Another joint from the same group could not be measured arthrometrically as the femur fractured when it was inserted into the arthrometer. The sample was subjected to the subsequent examinations. Incomplete decalcification meant that one sample from the control group intended for pathological examination had to be excluded. Two samples intended for qPCR analysis were excluded because they were not stored in accordance with the protocol.

### 3.2. Biomechanical Evaluation

#### 3.2.1. Assessment of Physiological Extension Deficit

A knee joint angle (JA) is defined by the intersection of the femur’s longitudinal axis and a line from the tibial plateau center to the upper ankle joint. Full geometric extension (fgE) is represented by a 180° joint angle but is restricted by the posterior joint capsule and the posterior cruciate ligament. A physiological extension deficit (pED) is the difference between fgE and the full physiological extension angle (pEA). In healthy rat knee joints, a torque of 35 Nmm achieves full physiological extension without capsular or ligamentous damage. For the placebo group of uninjured left knees, the pED was calculated as 30°, with an average extension angle of 150° (±20.6°) under 35 Nmm torque (Figure 3).

#### 3.2.2. Effect of Bosentan on Biomechanics

It appears that ET-1 levels correlate positively with the severity of fibrosis [29]. High levels of ET-1 in synovial fluid have also been found in inflammatory and fibrotic joint diseases, suggesting a potential link here as well [11,30]. To evaluate the antifibrotic efficacy of bosentan on joint biomechanics, the extension angle of the knee joint due to posterior joint capsule contracture (CA) was examined two weeks after trauma. At this point in time, preliminary studies have already shown a stable joint contracture after knee injury in a rat model [27,31,32].

Two weeks post-trauma, the rEA was determined at 35 Nmm of torque. The CA was then calculated by subtracting the rEA from the pEA (CA = pEA − rEA) (Figure 3A). Bosentan treatment resulted in a CA of 86.8° ± 14.1°, compared to 84.8° ± 11.1° in the placebo group, indicating no significant improvement in joint mobility (n.s., Mann–Whitney U test) (Figure 4A). Dynamic arthrometry assessed the force required to move the JA from 60° to 120°. It was quantified by calculating the area under the curve representing the force exerted by the automated arthrometer and the corresponding extension angle. The force applied by the arthrometer was calculated by multiplying the amperage used by a device-specific force constant (1N = 12 × 10 mA). Bosentan did not reduce the force needed to extend the joint from 60° to 120° (Figure 4B). As anticipated, the healthy knees, which served as a control group, exhibited a minimal requirement of force for joint extension when compared to the bosentan and placebo groups. For the latter two groups, this confirms the formation of a joint contracture. The total force required to extend the joint from 60° to 120° was calculated for each group (area under the curve). The placebo group exhibited a force 24 times greater than that of the control group.

### 3.3. Gene Expression Analysis of the Posterior Joint Capsule

**Figure 5:**
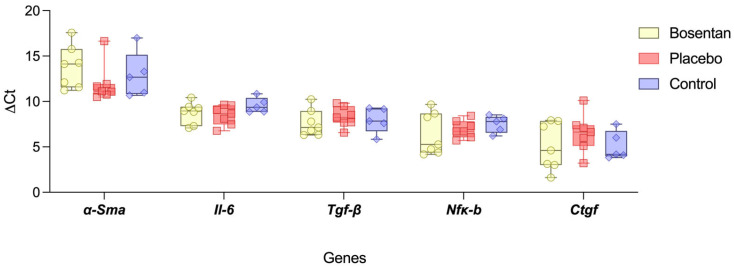
Gene expression of profibrotic upstream mediators. The box plot compares the gene expression levels (ΔCt) of *Il-6*, *Tgf-β*, *Nfκ-b*, and *Ctgf* of the bosentan (*n* = 7), the placebo (*n* = 7), and the uninjured control groups (*n* = 5). *Gapdh* is used for gene normalization. The minimum and maximum are indicated by the whiskers; the bar indicates the median. No statistical significance could be found between the groups, indicating a completed inflammatory phase (n.s., one-way ANOVA, post hoc Tukey test).

The expression levels of profibrotic mediators and their downstream effector genes were analyzed in the posterior joint capsules two weeks post-trauma, normalized to the housekeeping gene Gapdh. For the selected genes, including *Il-6*, *Tgf-β*, *Nfκ-b*, *Ctgf*, and *α-Sma* no significant differences were observed between the bosentan group and the placebo group. Two weeks after trauma, however, there was also no measurable difference between the healthy knees of the control group and the aforementioned groups (n.s., one-way ANOVA, post hoc Tukey test) (Figure 5).

### 3.4. Pathohistological Evaluation

Immunohistochemical analysis identified *α-Sma*-positive myofibroblasts, distinguishing them from endothelial cells, smooth muscle cells, and *α-Sma*-negative fibroblasts. A total of nine HPFs across three slides per specimen were evaluated by two independent investigators. The average was calculated. No measurable difference was observed in the absolute myofibroblast counts between the bosentan and placebo groups (bosentan = 30.2 ± 8.8 vs. placebo 20.7 ± 11.8). However, a significant difference was observed when the bosentan (bosentan = 30.2 ± 8.8 vs. control 1.0 ± 8.9, *p* < 0.001) or the placebo (placebo = 20.7 ± 11.8 vs. control 1.0 ± 8.9, *p* < 0.01; one-way ANOVA, post hoc Tukey test) group was compared to the control group.

**Figure 6:**
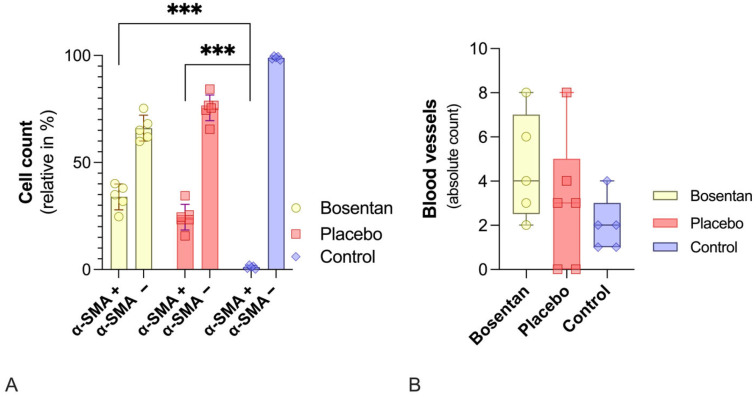
Histological evaluations of the posterior joint capsules. (**A**) Bar chart of the relative number of *α-Sma*(+) and *α-Sma*(−) cells. Bosentan (*n* = 5) has no effect on myofibroblast numbers compared to the placebo group (*n* = 6). Healthy knee joints in the control group show virtually no myofibroblasts. Significant differences are indicated by *** (*p* ≤ 0.001, one-way ANOVA). (**B**) Illustrates the absolute number of vessels. No statistically significant differences between the bosentan, placebo, and control groups were revealed (one-way ANOVA, post hoc Tukey test).

For better comparability, the number of *α-Sma*(+) cells was normalized based on the total number of cells. Again, there was no significant difference between the bosentan and placebo groups (30.2% ± 8.8% vs. 20.6% ± 11.8%, n.s., one-way ANOVA, post hoc Tukey test). Both groups differ significantly from the control group, in which only 1.0% ± 0.8% of cells were myofibroblasts (Figure 6A). In the control group, these *α-Sma*(+) cells are most likely artifacts.

The average number of blood vessels in the same HPFs was also determined. No relevant difference was found between the three groups (Figure 6B).

## 4. Discussion

After joint surgery and trauma, PTJC remains a challenging and difficult-to-treat complication. The limited therapeutic options, consisting of mechanical interventions of varying invasiveness, often lead to unsatisfactory results [3,4]. Currently, an effective pharmacological approach for PTJC is lacking, representing a significant unmet medical need. An ideal pharmacological treatment should have the potential to either prevent or limit the formation of PTJC. Among the various possible targets for correcting dysregulated profibrotic signaling cascades, the endothelin (ET) pathway emerged as a promising candidate. Apart from its vasoactive effects, ET-1 plays a pivotal role in the development of various forms of fibrosis [33,34]. Bosentan is a dual endothelin receptor antagonist and stands out as the most prominent candidate in its class [14,34]. The agent has already demonstrated its antifibrotic effect in various animal studies [35,36,37,38]. Elevated synovial fluid and serum levels of ET-1 in other inflammatory joint diseases, such as osteoarthritis and rheumatoid arthritis, suggest its potential role in arthrofibrosis [11,30]. Therefore, the purpose of this study was to evaluate the antifibrotic efficacy of orally administered bosentan on the development of PTJC in the surgically traumatized knee joints of 26 male Sprague Dawley rats over a total period of two weeks.

Contrary to findings in other fibrotic diseases, bosentan showed no measurable efficacy in preventing the formation of early-onset PTJC at either the biomechanical or pathohistological level.

The duration of pharmacological intervention of two weeks was based on our preliminary work on the formation of PTJC, as well as the changes in myofibroblast number and ET-1 expression levels described in the literature, and the established dosing strategies of the drug [17,18,19]. In our PTJC model, a biomechanically measurable knee joint contracture was already confirmed within a two-week immobilization phase. The contracture can be primarily attributed to the posterior knee joint capsule [24]. Intriguingly, even doubling the immobilization period did not produce any mechanically measurable increases in the severity of the contracture in our previous studies [27,31,32]. At two weeks, myofibroblast differentiation, the main effector cells of PTJC, is most pronounced [32,39]. With regard to ET-1, it can be assumed that expression levels are particularly high during the inflammatory phase [40]. Morrey et al. observed >100 inflammatory genes in their work on the molecular landscape of PTJC in rabbits over a period of 2 weeks. The expression levels of pro-inflammatory genes returned to baseline after 2 weeks [8,30]. They identified IL-1 as an early inductor for ET-1. Its expression level peaked within the first 24 h after trauma and returned to baseline after 72 h [8]. Our model confirmed that the inflammatory phase of PTJC had already resolved over a period of two weeks. The expression levels of the pro-inflammatory cytokine IL-6 and transcription factor NF-kB were comparable in the placebo and control groups (Figure 5). Interestingly, Kong et al. were able to prove that NF-kB plays a central role in the formation of PTJC. The injection of RelA7p65, an essential component of NF-kB, led to a significant increase in joint contracture in traumatized and immobilized rat knee joints [41].

The inflammatory phase may end well before this time in our model, as *α-Sma* levels were also comparable in both groups. Increased levels indicate a differentiation of fibroblasts into myofibroblasts [42,43]. The dose of bosentan used (100 mg/kg/day) is comparatively high compared to the doses used in the literature for fibrotic studies in rodents [44,45,46].

The following aspects could explain PTJC’s lack of response to bosentan. Regarding pharmacokinetics, the plasma concentration of bosentan peaks at 3–5 h after oral administration . This is in line with the observations of Iglarz et al. who found that a single oral dose of bosentan achieved maximum efficacy after 6 h in a rat model of bleomycin-induced pulmonary hypertension [47]. Although exact data on plasma concentrations after repeated bosentan administration for rodent models are currently unavailable, it can be assumed that a steady state is eventually reached after 3–5 days [48]. It is possible that the concentration of ET-1 was already at a peak level by this time. It remains to be evaluated whether bosentan therapy must be initiated before trauma, e.g., elective knee surgery, in order to achieve an antifibrotic effect. Future experimental setups could further elucidate the influence of this mechanism on PTJC by using qPCR, Western blot for the quantification of ET-1, or immunochemical staining of the ET-1 receptor.

Although increased synovial ET-1 concentrations have been demonstrated in both fibrotic and inflammatory joint diseases, the complex nature of the profibrotic signaling cascades, with their many bypass pathways, may limit the role of isolated inhibition of the endothelin axis in the treatment of PTJC [11,30]. Substance P, a neuropeptide with well-established pro-inflammatory properties, was also found at elevated levels in the tissue of arthrofibrotic knees [49,50]. Contrary to the expected antifibrotic effects of neurotransmitter inhibition, Morrey et al. found that in addition to the upregulation of anti-inflammatory mediators, substance P inhibition resulted in compensatory increases in Il-6 and Il-8 among other profibrotic mediators in their PTJC model [51]. Therefore, targeting multiple fibrotic pathways might be more efficient to limit PTJC formation. This could be achieved by antifibrotic drug combinations or agents with an affinity for multiple profibrotic pathways [52,53]. Furthermore, the selective inhibition of individual profibrotic cascades carries the risk of an unfavorable risk–benefit profile due to the dose-limiting toxicity of a single drug [54].

It is also possible that the agent’s lack of antifibrotic efficacy is due to insufficient bioavailability. The oral co-administration of white chocolate cream could have had a negative effect on bosentan’s serum concentration. However, the likelihood of the vehicle having an influence on the serum concentration of bosentan is low, as bioavailability studies have not revealed any impact when the drug is taken with food [55]. Rather, the fatty milieu of the chocolate cream may have a positive effect on the absorption of the lipophilic agent and has already been administered in a similar formulation in other animal studies [56,57]. An increase of the bioavailability directly in the joint could be achieved, for example, by repeated intra-articular injections. However, the rationale behind the chosen oral dosage form is its practical implementation in everyday clinical practice. Intra-articular injections are particularly associated with complications such as pain, bleeding, and infection, and are tedious for patients and medical staff alike [58]. Ultimately, this could have a negative impact on patient compliance.

## 5. Conclusions

Although preclinical evidence from both cellular and animal models suggests that endothelin-1 (ET-1) is a key mediator of tissue fibrosis, our findings indicate that it plays only a limited role in the pathogenesis of early onset post-traumatic joint contracture (PTJC). In our comprehensive, multidimensional animal study evaluating prophylactic bosentan therapy for PTJC, no significant antifibrotic effects were observed at the biomechanical, histological, or molecular level. These results support the emerging view that targeting a narrow subset of profibrotic signaling pathways, such as the endothelin axis, may be insufficient to prevent the complex and multifactorial development of PTJC.

## Figures and Tables

**Table 1:** Genes and primer sequences used for qPCR.

Gene	Primer	Sequence
*Gapdh*	Forward	AACGACCCCTTCATTGACCT
	Reverse	CCCCATTTGATGTTAGCGGG
*α-Sma*	Forward	CATCATGCGTCTGGACTTGG
	Reverse	CCAGGGAAGAAGAGGAAGCA
*Il-6*	Forward	CCACCCACAACAGACCAGTA
	Reverse	ACTCCAGAAGACCAGAGCAG
*Tgf-β1*	Forward	CCCTACATTTGGAGCCTGGA
	Reverse	CGCACGATCATGTTGGACAA
*Nfκ-b*	Forward	AGAGGATGTGGGGTTTCAGG
	Reverse	GCTGAGCATGAAGGTGGATG
*Ctgf*	Forward	TCCCAAAATCTCCAAGCCTA
	Reverse	GTAATGGCAGGCACAGGTCT

## Data Availability

The datasets generated and analyzed during the current study are available from the corresponding author upon reasonable request.

## Abbreviations

The following abbreviations are used in this manuscript:

**Abbreviation**	**Meaning**
PTJC	post-traumatic joint contracture
CA	contracture angle
*Col1a1*	Collagen, Type I, Alpha 1
*Ctgf*	connective tissue growth factor
ET-1	endothelin-1
ETAR	endothelin recptor type a
ETBR	endothelin recptor type b
fgE	full geometric extension
*Gapdh*	glyceraldehyde-3-phosphate dehydrogenase
HPF	high-power field
*Il-6*	interleukin 6
JA	joint angle
*NFκ-B*	nuclear factor ‘kappa-light-chain-enhancer’ of activated B-cells
pEA	physiological extension angle
pED	physiological extension deficit
rEA	restricted extension deficit
*Tgf-β1*	transforming growth factor beta1
*α-Sma*	alpha-smooth muscle actin
